# Trajectories of Psychopathology According to Continuation or Discontinuation of Child Abuse: A Longitudinal Observational Study

**DOI:** 10.3390/ijerph18178968

**Published:** 2021-08-26

**Authors:** Eunji Jung, Joung-Sook Ahn, Jaehyun Han, Min-Hyuk Kim

**Affiliations:** Department of Psychiatry, Yonsei University Wonju College of Medicine, 20 Ilsan-ro, Wonju 26426, Gangwon-do, Korea; ejy8676@yonsei.ac.kr (E.J.); jsahn@yonsei.ac.kr (J.-S.A.)

**Keywords:** child abuse, maltreatment, discontinuation, psychopathology, externalizing, internalizing, longitudinal observation

## Abstract

Long-term exposure to childhood abuse and occurrence of mental illness are positively correlated. Using long-term tracking data in Korea, we identified the characteristics of children and adolescents who experienced abuse and impact thereof on their psychopathology. Using the Korea Welfare Panel data, 354 teenagers in grades 4–6 of elementary school participated, were assessed at baseline, and monitored 3 years later. They were categorized into Never, Occurrence, Continuation, and Discontinuation groups according to changes in the abuse experienced. Psychopathology was evaluated using K-CBCL. Childhood abuse experience significantly affected psychopathology. At the baseline, the Continuation and Discontinuation groups had a higher severity of psychopathology than the Never group. Psychopathology at the baseline was associated with whether the patient experienced abuse that year. In the follow-up observation, the risk of psychopathology in the Occurrence and Continuation groups was higher than that in the Never group. The Discontinuation group had decreased psychopathology, which was not clinically significant in the follow-up observation (INT aRR = 2.09; 95% CI 0.61–7.13, EXT aRR = 4.23; 95% CI 1.12–16.07). Stopping abuse in late childhood reduces adolescents’ psychopathology in the long term, meaning they can recover their normal developmental trajectory according to risk groups and provide effective interventions including discontinuation of abuse.

## 1. Introduction

Recent neurobiological research has explored the development of the human brain during childhood and adolescence. This is a critical time in neurological development, and experiences of childhood abuse including physical, sexual, and emotional abuse and neglect, lead to children and adolescents deviating from their normal developmental trajectories. Children who experience abuse have a high risk of developing externalizing symptoms such as aggression and antisocial behavior in adolescence [1,2]. Abused children are also at high risk of developing internalizing symptoms, such as depression and anxiety, low self-esteem, and interpersonal problems [3]. Abuse increases psychopathology in childhood and adolescence, which can lead to various mental illnesses in adulthood [4,5,6].

There is a clear positive correlation between the duration of exposure to childhood abuse experiences and the incidence of psychopathology. In particular, the risk of persistent and repeated abuse leading to psychopathology is high [7,8,9]. While several studies have noted the effects of chronic abuse on psychopathology, few long-term follow-up studies using representative samples have been conducted [10]. Unlike previous longitudinal studies that observed functional trajectories in general, few studies have focused on whether the discontinuation of abuse reduces its effect on psychopathology [11]. A study was needed to confirm that psychopathology can be mitigated if abuse is discontinued, which was the aim of the current study. If discontinuation of child abuse could significantly reduce the development of psychopathology, this study would provide important evidence for prioritizing child abuse interventions.

The number of child abuse reports in Korea has steadily increased every year, with a total of 30,045 cases of domestic child abuse in 2019 [12]. Given this situation, a long-term follow-up study is needed to confirm the impact of childhood abuse experiences on psychopathology. We used the Korea Welfare Panel (KOWEPS) data—the representative sample data released in Korea—to (1) identify the characteristics of children and adolescents who have experienced abuse, and (2) analyze the long-term effects of their abuse experiences.

## 2. Methods

### 2.1. Data and Participants

In this study, the baseline (taken in 2015) and a 3-year follow-up (done in 2018) of household data and children’s survey data from the KOWEPS (Korea Welfare Panel Study; www.koweps.re.kr, accessed on 14 March 2020) were combined and analyzed. The Korea Welfare Panel is a long-term survey conducted annually by the Korea Institute of Social Health and Seoul National University Social Welfare Research Institute, which includes representative samples of approximately 7000 households nationwide. The target population of KOWEPS concerns all households living across the country, representing 90% of the census. Trained interviewers visit participants’ homes and collect data through interviews. Additional surveys of children younger than 15 years are conducted every 3 years. In this study, 354 adolescents from grades 4–6 of elementary school were selected at baseline to check the longitudinal relationship of psychopathology according to the changes in abuse experiences. The mean (SD) age of the participants was 10.05 (0.799) years; 167 (47.2%) were male and 187 (52.8%) were female. Among the participants, 12 (3.4%) used alcohol, 3 (0.8%) smoked, and 48 (13.6%) had chronic diseases. A total of 337 (95.2%) were living with both parents, while 17 (4.8%) were living with single parents or grandparents. The household income of 304 (85.9%) participants was below the 60th percentile, and 23 (6.5%) participants received basic livelihood security. The study also employed data tracked 3 years later when they were in grades 1–3 of middle school.

### 2.2. Child Abuse

Previous research has shown that emotional abuse or neglect significantly impacts an individual and cause greater vulnerability to mental illness, even at minor levels, regardless of type [13]. Therefore, in this study, we investigated the association between psychopathology according to the accumulation and changes in the experience of abuse itself, rather than the severity or type of abuse.

Of the four total abuse experience questions, which consist of one physical abuse question and three emotional abuse questions, “abuse” is indicated if any question applies, and “no abuse” is indicated if none of the four questions apply. Answering the question “I have been severely beaten by my parents” pertaining to occurrences over the past year has been considered as experiencing physical abuse (PA). Emotional abuse (EA) consists of three questions pertaining to occurrences over the past year, for example: “I was scolded by parents enough to feel ashamed and insulted,” and “My parents have told me such things as ‘I would feel better without you,’ ‘You are a fool,’ or ‘You are a worse person than a dog.’” The Cronbach’s α for the four questions consisting of one physical abuse and three emotional abuse questions was 0.808 (baseline) and 0.757 (follow-up), respectively.

### 2.3. Group Classification

The four groups were delineated according to changes in the abuse experiences over time. The control group (Never; N) reported no abuse at baseline and at follow-up; the Occurrence group (O) did not report abuse at baseline but reported abuse at follow-up, the Continuation group (C) reported abuse at baseline and at follow-up, and the Discontinuation group (D) reported abuse at baseline but no abuse at follow-up.

### 2.4. Psychopathology

To assess adolescents’ psychopathology, we used the standardized Child Behavior Checklist in Korean (K-CBCL) [14,15,16]. It consists of 25 questions on internalizing symptoms (14 questions on depression and anxiety and 11 on attention-concentration) and 41 questions on externalizing symptoms (9 questions on withdrawal, 13 on delinquency, and 19 on aggression). The Cronbach’s α of the K-CBCL is 0.62–0.86 [17,18]. The K-CBCL original score was converted to a T-score using the average and standard deviation of each group divided by the age and gender of the Korean Welfare Panel data. We set the cutoff point that distinguished the clinical significance to 64 points corresponding to the 2 SD.

### 2.5. Statistical Analysis

To identify the socio-demographic characteristics of the target population, a comparison was performed using a χ^2^ test. Moreover, ANOVA was used to compare the mean values of the K-CBCL scores according to whether the adolescents were abused at baseline and follow-up, and the Bonferroni method was employed as a post-test. Logistic regression analysis was used to determine the effect of longitudinal changes in abuse on each of the internalizing and externalizing symptoms at the follow-up point. In the logistic regression analysis, three models were used for covariate correction. The first model was a univariate analysis, and the second was a multivariate analysis including age, sex, academic achievement, alcohol use, smoking, chronic disease, cohabitation, family income, and basic livelihood security. In the third model, the presence of psychopathology in the K-CBCL that was investigated at baseline was used as an additional covariate. It was used to assess the effects of changes that abuse experiences had on psychopathology at the follow-up point. All analyses were conducted using IBM-SPSS 25.0, and schematized using the ggplot2 package of R.

## 3. Results

### 3.1. Socio-Demographic Characteristics

At baseline, 102 (28.8%) participants reported experiencing abuse over the past year, and 38 (10.7%) experienced both types of abuse. Furthermore, 58 (16.4%) reported experiencing physical abuse and 82 (23.2%) reported emotional abuse.

Table 1 shows the difference in composition ratio according to age, sex, academic achievement, alcohol use, smoking, chronic disease, cohabitation, family income, and basic livelihood security of the 354 participants. As a result of conducting a χ2 test to determine the statistical significance of abuse by each factor, the abuse experienced was significant according to the difference in academic achievement (*p* = 0.02). However, there were no significant differences between the groups according to age, sex, alcohol use, smoking, chronic disease, cohabitation, family income, and basic livelihood security.

### 3.2. Comparison of Psychopathology between Groups According to Changes in Abuse Experiences

After classifying the groups according to changes in abuse experiences, the K-CBCL score averages of the four groups were compared to determine each group’s psychopathological characteristics (Table 2). Comparing K-CBCL scores at baseline, the Bonferroni post-test showed that the severity of psychopathology in the Continuation (C) and Discontinuation groups (D) was higher on average than that of the Never group (N) (C: INT *p* = 0.005, EXT *p* = 0.001; D: INT *p* = 0.001, D: EXT *p* = 0.004). The level of psychopathology at baseline was associated with whether abuse was experienced in the past year. The severity of internalizing or externalizing symptoms could not predict whether abuse would continue in the future.

### 3.3. Short-term Effects of Childhood Abuse Experiences

To distinguish clinical significance, we set the K-CBCL cutoff point to 64 points corresponding to the 2 SD. A logistic regression analysis was conducted to determine the short-term effect of abuse on psychopathology in the same year at baseline (Table 3). During the analysis, the effects of age, sex, academic achievement, alcohol use, smoking, chronic disease, cohabitation, family income, and basic livelihood security were corrected. The analysis showed that experiencing abuse had a significant effect on psychopathology (INT aOR = 3.19, EXT aOR = 3.13; both *p* < 0.01).

### 3.4. Long-Term Effects of Childhood Abuse Experiences

**Figure 1:**
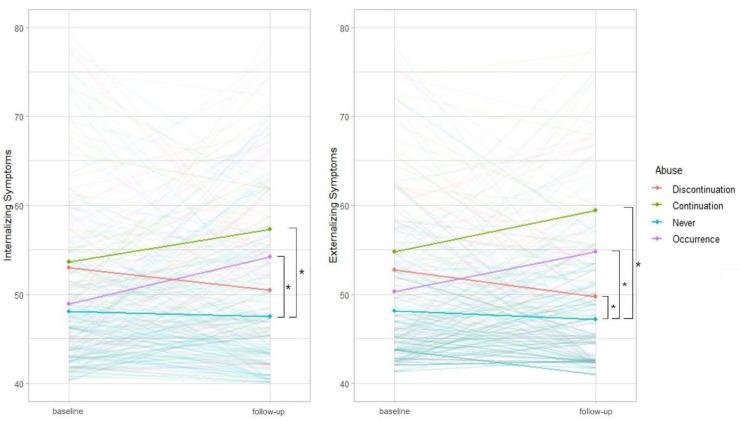
Effect of variation of abuse on internalizing/externalizing symptoms at follow-up. *: Significant relative risk. At baseline, the risk of psychopathology in the Continuation and Occurrence groups significantly increased. The K-CBCL score in the Discontinuation group decreased. Compared to those in the Never group, the internalizing symptoms of the Discontinuation group were no longer significant. Externalizing symptoms decreased, but remained significant.

A logistic regression analysis was conducted to determine the effect of changes in abuse experiences on follow-up K-CBCL scores and the clinical significance of psychopathology (Table 4, Figure 1). Model 3, which corrected K-CBCL scores at baseline, also showed that both the Occurrence group (O) and Continuation group (C) had a significantly higher risk of psychopathology at follow-up than the control group (N) (O: INT aRR = 9.36, EXT aRR = 14.93; C: INT aRR = 6.29, EXT aRR = 16.41; all *p* < 0.01).

The K-CBCL scores in the Discontinuation group (D) decreased (Figure 1) and compared with the controls, the effects on the clinical risk of psychopathology varied depending on the type of internalizing or externalizing symptoms. Internalizing symptoms were no longer significant (aRR = 2.09; 95% CI 0.61–7.13; *p* = 0.24), and externalizing symptoms remained significant, but had decreased significantly (aRR = 4.23; 95% CI 1.12–16.07; *p* = 0.03). The clinical risk of psychopathology of the Occurrence (O) and Continuation group (C) increased significantly in both internalizing and externalizing symptoms (O: INT aRR = 9.36; 95% CI 3.20–27.40, EXT aRR = 14.93; 95% CI 4.26–52.40; C: INT aRR = 6.29; 95% CI 1.93–20.48, EXT aRR = 16.41; 95% CI 4.64–58.00; all *p* < 0.01), while the Never group (N) showed no significant difference. The subgroup analysis showed that unlike physical abuse, externalizing symptoms no longer exhibited clinical risk compared to the controls, especially when emotional abuse was discontinued (INT aRR = 2.34; 95% CI 0.76–7.22; *p* = 0.14, EXT aRR = 3.15; 95% CI 0.93–10.64; *p* = 0.06).

In contrast, the risk of internalizing symptoms and externalizing symptoms increased 9.36 times and 14.93 times respectively in the Occurrence (O) group than in the control group (INT aRR = 9.36, EXT aRR = 14.93; both *p* < 0.01). For the Continuation group (C), the risk of internalizing symptoms and externalizing symptoms increased 6.29 times and 16.41 times respectively (INT aRR = 6.29, EXT aRR = 16.41; both *p* < 0.01).

## 4. Discussion

This study analyzed the relationship between childhood abuse experiences and early adolescents’ psychopathology using representative domestic samples. The group that reported continuous abuse between the baseline and follow-up period demonstrated a high incidence of psychopathology 3 years later compared to the non-abuse groups. The group that reported abuse at baseline but reported the discontinuation of abuse in the follow-up did not differ in the level of psychopathology as compared to the Continuation group at baseline; however, its severity decreased significantly after the discontinuation of abuse. In contrast, the Occurrence group did not differ in the level of psychopathology from the Never group at baseline, but the severity increased significantly after the abuse occurred. The results were the same regardless of the type of abuse when analyzing subgroups that distinguished between emotional and physical abuse.

### 4.1. Acute and Chronic Effects of Childhood Abuse Experiences

Abuse at the baseline had a significant effect on psychopathology in the same year. When groups were delineated based on whether abuse continued through the follow-up observation, the group experiencing continued abuse was already at a higher risk of psychopathology at the base compared to the group that did not experience abuse. This is consistent with the existing research on the acute effects of childhood abuse experiences [3].

These results suggest that psychopathology at the age of 10–12 years may be an indicator of recent abuse experiences. For children aged 10–12 years, experiencing abuse causes changes in the structure of their medial temporal lobes, which can lead to internalizing symptoms such as depression, anxiety, and attention deficit [19]. In addition, abused children may also have interpersonal conflicts due to aggressive behavior [7,20,21] and are often rejected by peers at a time when socialization is important, thus further aggravating externalizing symptoms [22,23].

Meanwhile, it is not clear whether the psychopathology of this period is the result of only recent experiences of abuse. Reportedly, psychopathology in children who experience abuse before age 4 is evident after the age of 10–12 years [24,25]. The earlier the abuse begins and the longer the total duration of exposure to it, the worse is the child’s functional development trajectory [8]. Psychopathologies that emerged in early adolescence may be influenced by recent abuse, as well as cumulative abuse experiences from an earlier age. Although there are several studies on the temporal causal relationship between abuse experiences and manifestation of psychopathology [7,26], there are insufficient results that conclusively support either acute or chronic effects of abuse [25]. Therefore, further research is needed to identify the temporal effects of abuse according to the developmental period of children.

### 4.2. Discontinuation of Childhood Abuse and Changes in Psychopathology

The study identified the long-term effects of the discontinuation of abuse, which contributes to reducing psychopathology. Follow-up observations showed a significant reduction in the risk of psychopathology, as abuse was discontinued even after adjusting for underlying psychopathology.

The risk of psychopathology was significantly higher when experiencing continuous abuse than when there was no or discontinued abuse. This is consistent with previous research showing that children who experience chronic abuse are at a higher risk of developing various psychopathologies than those who experience temporary abuse [10,25]. Children who experience frequent abuse exhibit more aggressive behavior, which often leads to interpersonal conflicts [7]. The longer the total duration of exposure to abuse, the worse the child’s functional trajectory and the higher the risk of psychopathology. This means that there is an association between the amount of time children are exposed to abuse and psychopathology [8].

The study identified a significant association between the discontinuation of abuse and a marked reduction in psychopathology. This shows that active intervention including the discontinuation of abuse in children aged 10–12 years, can be a key way to protect children from the risk of psychopathology. This result is consistent with existing long-term follow-up studies suggesting that the discontinuation of abuse reduces the effects on psychopathology [11,25].

In this study, when abuse was discontinued, the internalizing symptoms decreased to an indistinguishable extent from those in the non-abuse group, but externalizing symptoms remained significant despite the decrease. Similarly, a long-term follow-up study found that internalizing symptoms in abused children decreased to an indistinguishable level after mental health service intervention, but externalizing symptoms remained significant [27]. Thus, it can be assumed that internalizing symptoms respond relatively well to intervention, while externalizing symptoms respond less to treatment.

Later childhood (children aged 10–12 years), which is the basis of this study, is cited as a critical time during brain development. Recent neurobiological studies have noted the responsiveness of the amygdala to abusive experiences [28]. In particular, the right amygdala has been reported to be vulnerable to minor abuse such as verbal abuse and neglect between the ages of 10 and 11 years [29].

The clinical significance of the study was that the discontinuation of abuse in children aged 10–12 years, an age suitable for monitoring, screening, and intervention, confirmed the possibility of reducing psychopathology in early adolescents aged 13–15 years.

## 5. Limitations and Implications

There are several limitations to this study.

First, a relatively small sample of 354 children and adolescents was analyzed. However, it has sufficient clinical significance because it uses long-term follow-up observations from representative samples. This study also reproduced statistically significant results using a larger number of samples than existing studies with similar designs [11].

Second, we established highly sensitive questions that distinguished between the presence and absence of abuse experiences. Further analysis in this study identified a positive correlation between psychopathology and abuse frequency, indicating that the association with the type of abuse was not significant. Although existing studies deal with the link between the severity of abuse and level of psychopathology, this study focused on quantitative relationships based on the accumulation of time exposed to abuse rather than the severity thereof. Existing studies have noted the major impact of the presence or absence of abuse on psychopathology, rather than the type, frequency, and severity of abuse when setting a cutoff for abuse experiences [13].

Third, the types of child abuse did not include neglect, sexual abuse, or witnessing domestic violence. Several existing studies include these types of abuse, such as Adverse Childhood Experiences (ACEs), and have found that neglect is especially as influential as all other types of abuse [4,13]. In this study data, the rate of participants who answered in the positive for the neglect and sexual abuse questions was extremely low. Therefore the results were heterogeneous from those who answered in the positive for physical and emotional abuse questions. The internal validity of abuse questions was reduced when neglect and sexual abuse questions were included, and the statistical analysis did not produce significant results. Appropriate questions to assess witnessing domestic violence were impossible to find within the data. To extend the results of this study to other important types of abuse, additional follow-up studies are needed, including data that can adequately evaluate neglect, sexual abuse, and witnessing domestic violence.

Fourth, there may be confounders that were not considered in this study. In particular, the effects of protective factors have not been sufficiently controlled. Existing studies have reported that individuals’ inner traits, such as positive self-esteem, self-resilience, and control, are protective factors that predict resilience [30,31]. Although some protective factors have been found to have a universal effect on all children with or without abuse [32], childhood abuse experiences are independent predictors that show special trajectories to form subtypes within the same psychopathological group [33]. Additional follow-up studies are needed to identify specific protective factors for children who experience abuse.

Fifth, as the participants’ races and ethnicities are homogeneous, this study may be limited in generalizing to racial and ethnic groups beyond the Korean population.

Despite these limitations, this study has several important implications. Representative samples were monitored for a long period, confirming the possibility that psychopathology in late childhood could suggest an abuse experience. This suggests that the previous abuse experiences of children who present psychopathology at the time of evaluation in late childhood must be intensively evaluated and interventions should be implemented. The study also showed the possibility that the discontinuation of abuse thereof could reduce psychopathology in adolescence in the long term. This suggests that the prevention of child abuse requires screening of risk groups and active, specialized interventions, especially as direct abuse discontinuation can restore adolescents’ developmental trajectory.

## Figures and Tables

**Table 1:** Socio-demographic characteristics of participants at baseline.

		Total (N = 354)	Abuse (N = 102)	No Abuse (N = 252)		Subgroup Analysis					
						PA (N = 58)	No PA (N = 296)		EA (N = 82)	No EA (N = 272)	
		n(%)	n(%)	n(%)	*p*	n(%)	n(%)	*p*	n(%)	n(%)	*p*
Age (mean (SD))		10.05 (0.799)	10.10 (0.827)	10.02 (0.788)	0.44	9.97 (0.837)	10.06 (0.792)	0.43	10.03 (0.791)	10.10 (0.826)	0.51
Sex	Male	167 (47.2%)	53 (52.0%)	114 (45.2%)	0.25	34 (58.6%)	133 (44.9%)	0.06	44 (53.7%)	123 (45.2%)	0.21
	Female	187 (52.8%)	49 (48.0%)	138 (54.8%)		24 (41.4%)	163 (55.1%)		38 (46.3%)	149 (54.8%)	
Academic achievement	High	22 (6.2%)	11 (10.8%)	11 (4.4%)	0.02 *	7 (12.1%)	15 (5.1%)	0.10	9 (11.0%)	13 (4.8%)	0.08
	Middle	254 (71.8%)	75 (73.5%)	179 (71.0%)		41 (70.7%)	213 (72.0%)		59 (72.0%)	195 (71.7%)	
	Low	78 (22.0%)	16 (15.7%)	62 (24.6%)		10 (17.2%)	68 (23.0%)		14 (17.1%)	64 (23.5%)	
Alcohol use	No	342 (96.6%)	100 (98.0%)	242 (96.0%)	0.35	57 (98.3%)	285 (96.3%)	0.70	80 (97.6%)	262 (96.3%)	0.74
	Yes	12 (3.4%)	2 (2.0%)	10 (4.0%)		1 (1.7%)	11 (3.7%)		2 (2.4%)	10 (3.7%)	
Smoking	No	351 (99.2%)	102 (100.0%)	249 (98.8%)	0.27	58 (100.0%)	293 (99.0%)	1.00	82 (100.0%)	269 (98.9%)	1.00
	Yes	3 (0.8%)	0 (0.0%)	3 (1.2%)		0 (0.0%)	3 (1.0%)		0 (0.0%)	3 (1.1%)	
Chronic disease	No	306 (86.4%)	89 (87.3%)	217 (86.1%)	0.78	49 (84.5%)	257 (86.8%)	0.67	72 (87.8%)	234 (86.0%)	0.85
	Yes	48 (13.6%)	13 (12.7%)	35 (13.9%)		9 (15.5%)	39 (13.2%)		10 (12.2%)	38 (14.0%)	
Cohabitants (caregiver)	Both parents	337 (95.2%)	99 (97.1%)	238 (94.4%)	0.30	55 (94.8%)	282 (95.3%)	0.75	80 (97.6%)	257 (94.5%)	0.38
	Single parent /Grandparents	17 (4.8%)	3 (2.9%)	14 (5.6%)		3 (5.2%)	14 (4.7%)		2 (2.4%)	15 (5.5%)	
Family income	<60th percentile	304 (85.9%)	85 (83.3%)	219 (86.9%)	0.38	48 (82.8%)	256 (86.5%)	0.54	68 (82.9%)	236 (86.8%)	0.37
	>60th percentile	50 (14.1%)	17 (16.7%)	33 (13.1%)		10 (17.2%)	40 (13.5%)		14 (17.1%)	36 (13.2%)	
Basic livelihood security	No	331 (93.5%)	94 (92.2%)	237 (94.0%)	0.51	52 (89.7%)	279 (94.3%)	0.24	76 (92.7%)	255 (93.8%)	0.80
	Yes	23 (6.5%)	8 (7.8%)	15 (6.0%)		6 (10.3%)	17 (5.7%)		6 (7.3%)	17 (6.3%)	

Note. PA, physical abuse; EA, emotional abuse. * Statistically significant results (*p* < 0.05).

**Table 2:** K-CBCL scores at baseline and follow-up by group according to the variation of abuse.

		Total Abuse				Subgroup Analysis							
						PA				EA			
		N	O	D	C	N	O	D	C	N	O	D	C
		N = 207	N = 45	N = 65	N = 37	N = 268	N = 28	N = 45	N = 13	N = 227	N = 45	N = 58	N = 24
		Mean (SD)	Mean (SD)	Mean (SD)	Mean (SD)	Mean (SD)	Mean (SD)	Mean (SD)	Mean (SD)	Mean (SD)	Mean (SD)	Mean (SD)	Mean (SD)
INT	Baseline	48.01 (8.21)	48.90 (8.41)	53.02 * (12.05)	53.68 * (11.83)	48.57 (8.83)	49.74 (9.17)	54.94 * (13.03)	52.90 (9.02)	48.08 (8.13)	50.15 (9.82)	53.98 * (12.52)	52.89 (11.89)
	Follow-up	47.49 (7.85)	54.24 * (10.98)	50.47 (9.45)	57.31 * (12.28)	48.62 (8.66)	55.48 * (12.72)	51.57 (9.57)	59.04 * (13.92)	47.75 (7.95)	54.79 * (11.59)	51.44 * (10.09)	57.63 * (12.44)
EXT	Baseline	48.09 (7.54)	50.35 (10.92)	52.79 * (11.99)	54.76 * (12.54)	48.89 (8.89)	49.87 (9.69)	55.36 * (13.05)	52.91 (9.33)	48.41 (7.93)	51.05 (12.77)	52.90* (12.26)	55.16 * (9.83)
	Follow-up	47.17 (6.23)	54.78 * (12.23)	49.74 (9.59)	59.44 * (13.66)	48.45 (8.06)	54.70 * (13.24)	53.04 * (12.59)	58.29 * (11.32)	47.39 (6.80)	56.15 * (12.44)	50.72 (10.43)	59.84 * (13.35)

Note. Int: internalizing symptoms; Ext: externalizing symptoms; PA: physical abuse; EA: emotional abuse; N: Never group; O: Occurrence group; D: Discontinuation group; C: Continuation group. * Statistically significant results of the Bonferroni post-test compared to the N group (*p* < 0.05).

**Table 3:** Logistic regression analysis between recent abuse and psychopathology at baseline.

		Unadjusted				Adjusted			
		INT		EXT		INT		EXT	
		OR	95% CI	OR	95% CI	OR	95% CI	OR	95% CI
Abuse	No	1 (ref)		1 (ref)		1 (ref)		1 (ref)	
	Yes	3.42	1.58–7.40	3.17	1.45–6.93	3.19 *	1.40–7.25	3.13 *	1.34–7.28
PA	No	1 (ref)		1 (ref)		1 (ref)		1 (ref)	
	Yes	3.29	1.60–6.76	2.95	1.45–6.00	3.89 *	1.84–8.24	2.86 *	1.38–5.94
EA	No	1 (ref)		1 (ref)		1 (ref)		1 (ref)	
	Yes	4.28	2.13–8.59	3.37	1.71–6.62	4.44 *	2.19–9.00	3.17 *	1.59–6.30

Note. Int, internalizing symptoms; Ext, externalizing symptoms; PA, physical abuse; EA, emotional abuse. Adjusted: Adjusted for variables (age, sex, academic achievement, alcohol use, smoking, chronic disease, cohabitation, family income, basic livelihood security). Relative risks and 95% confidence intervals are reported. * Statistically significant results (*p* < 0.05).

**Table 4:** Logistic regression analysis between variation of abuse and psychopathology at follow-up.

			Model 1				Model 2				Model 3			
			INT		EXT		INT		EXT		INT		EXT	
			RR	95% CI	RR	95% CI	aRR	95% CI	aRR	95% CI	aRR	95% CI	aRR	95% CI
Abuse		N	1 (ref)		1 (ref)		1 (ref)		1 (ref)		1 (ref)		1 (ref)	
		O	8.16 *	2.91–22.88	14.50 *	4.31–48.81	9.32 *	3.20–27.16	15.49 *	4.48–53.54	9.36 *	3.20–27.40	14.93 *	4.26–52.40
		D	2.38	0.73–7.77	5.16 *	1.41–18.90	2.26	0.67–7.62	4.36 *	1.15–16.51	2.09	0.61–7.13	4.23 *	1.12–16.07
		C	6.67 *	2.18–20.34	24.36 *	7.30–81.32	6.82 *	2.11–21.98	20.06 *	5.84–68.98	6.29 *	1.93–20.48	16.41 *	4.64–58.00
Subgroup Analysis	PA	N	1 (ref)		1 (ref)		1 (ref)		1 (ref)		1 (ref)		1 (ref)	
		O	9.29 *	3.52–24.49	6.54 *	2.36–18.15	9.94 *	3.51–28.17	6.59 *	2.21–19.64	9.75 *	3.41–27.88	6.72 *	2.22–20.31
		D	1.40	0.38–5.13	4.24 *	1.65–10.92	1.54	0.40–5.89	3.79 *	1.40–10.24	1.44	0.37–5.52	3.26 *	1.18–9.03
		C	8.72 *	2.37–32.08	8.72 *	2.37–32.08	9.28 *	2.34–36.77	8.46 *	2.15–33.25	9.40 *	2.37–37.37	6.58 *	1.58–27.45
	EA	N	1 (ref)		1 (ref)		1 (ref)		1 (ref)		1 (ref)		1 (ref)	
		O	6.92 *	2.63–18.23	11.92 *	4.14–34.33	7.40 *	2.71–20.24	11.69 *	3.93–34.78	7.45 *	2.71–20.45	10.97 *	3.65–32.96
		D	2.79	0.95–8.20	4.25 *	1.32–13.71	2.56	0.84–7.76	3.34	1.00–11.18	2.34	0.76–7.22	3.15	0.93–10.64
		C	4.84 *	1.37–17.14	22.10 *	6.94–70.34	4.95 *	1.34–18.38	19.00 *	5.74–62.93	4.43 *	1.17–16.78	15.17 *	4.42–52.09

Note. Int: internalizing symptoms; Ext: externalizing symptoms; PA: physical abuse; EA: emotional abuse; N: Never group; O: Occurrence group; D: Discontinuation group; C: Continuation group. Model 1: Unadjusted. Model 2: Adjusted for variables (age, sex, academic achievement, alcohol use, smoking, chronic disease, cohabitation, family income, and basic livelihood security) without baseline K-CBCL score. Model 3: Adjusted for variables (age, sex, academic achievement, alcohol use, smoking, chronic disease, cohabitation, family income, and basic livelihood security) with baseline K-CBCL score. * Statistically significant results (*p* < 0.05). Relative risks and 95% confidence intervals are reported. In Model 3, internalizing symptom scores of Abuse/PA/EA for the Discontinuation group and externalizing symptom scores of EA for the Discontinuation group were not significant.

## Data Availability

Publicly available datasets were analyzed in this study. This data can be found here: [https://www.koweps.re.kr] (accessed on 14 March 2020).
